# Antibody-dependent enhancement of coronaviruses

**DOI:** 10.7150/ijbs.96112

**Published:** 2025-02-03

**Authors:** Tao Tao, Lili Tian, Jiayi Ke, Chuxie Zhang, Maochen Li, Xiaolong Xu, Junfen Fan, Yigang Tong, Huahao Fan

**Affiliations:** 1 College of Life Science and Technology, Beijing University of Chemical Technology, Beijing 100029, China.; 2 Beijing Hospital of Traditional Chinese Medicine, Capital Medical University, Beijing 100010, China.; 3 Institute of Cerebrovascular Disease Research and Department of Neurology, Xuanwu Hospital of Capital Medical University, Beijing 100053, China.; 4 School of Life Sciences, Tianjin University, Tianjin 300072, China.

**Keywords:** Antibody-dependent enhancement, Coronavirus, Neutralizing antibody, Vaccination

## Abstract

The COVID-19 pandemic presents a significant challenge to the global health and the world economy, with humanity engaged in an extended struggle against the virus. Notable advancements have been achieved in the development of vaccines and therapeutic interventions, including the application of neutralizing antibodies (NAbs) and convalescent plasma (CP). While antibody-dependent enhancement (ADE) has not been observed in human clinical studies related to SARS-CoV-2, the potential for ADE remains a critical concern and challenge in addressing SARS-CoV-2 infections. Moreover, the causal relationship between ADE and viral characteristics remains to be clearly elucidated. Viruses that present with severe clinical manifestations of ADE have demonstrated the capacity to replicate in macrophages or other immune cells, or to alter the immunological status of these cells, which induces abortive infections characterized by systemic inflammation. In this review, we summarize experimental observations and clinical evidence concerning the ADE effect associated with coronaviruses. We critically examine the potential mechanisms through which coronaviruses mediate ADE, and propose strategies to mitigate this phenomenon in the context of viral infection treatment. Our aim is to offer informed recommendations for the containment of the COVID-19 pandemic and to strengthen the response to SARS-CoV-2, as well as to prepare for potential future coronavirus threats.

## 1. Introduction

Antibody-dependent enhancement (ADE) is a phenomenon that exacerbates disease progression following viral reinfection 1. In cases of ADE, pre-existing antibodies fail to provide protective antiviral immunity; rather, they facilitate viral entry and augment viral infection within host cells, leading to increased infectivity and virulence 1,2. This phenomenon has been documented in various viruses both *in vitro* and *in vivo* models 3, such as Dengue virus (DENV), which has been studied in P-388D1 mouse macrophage-like cells, BHK cells and Dengue 2 virus (D2V)-infected monkeys 4-6, 21 years of dengue surveillance in Thailand 7,8; Zika virus (ZIKV) in myeloid cell lines U937 and in mice 9,10; Feline Infectious Peritonitis Virus (FIPV) in macrophages from mice and cats both *in vitro* and *in vivo* 11-15. Similarly, ADE has been identified in certain coronaviruses, such as Severe Acute Respiratory Syndrome Coronavirus (SARS-CoV) across multiple human immune cell lines, Vero E6 cells, Raji B and Daudi cells, as well as in macaque and ferret models 16-24, Middle East Respiratory Syndrome Coronavirus (MERS-CoV) in human lung and cervical cells 25, as well as artificial models with pseudovirus of Severe Acute Respiratory Syndrome Coronavirus-2 (SARS-CoV-2) 26,27.

Dengue fever (DF) is regarded as a prototypical disease model for investigating the ADE phenomenon. In 1977, Scott Halstead was the first to associate ADE with the severe manifestations of dengue fever, which are induced by the dengue virus 28. Given the well-documented association between ADE and the severity of dengue shock syndrome, extensive and intensive research has been undertaken to explore ADE within the context of DENV infection 2. Typically, infection with DENV confers long-lasting or even lifelong immunity to the infected individual. However, sequential infections with different DENV serotypes, such as initial infection with DENV-4 followed by DENV-2, result in the production of neutralizing antibodies that are ineffective against the subsequent serotype. This phenomenon can lead to more severe secondary infections 3,29. Notably, there exists a specific concentration of antibodies that most effectively enhances DENV infection 30. It has been reported that suboptimal antibody levels result in a slight enhancement of infection, whereas elevated antibody titers are capable of effectively neutralizing viral particles, thus providing a safer outcome in the context of DENV infection 30. The pre-existing antibodies lacking neutralizing activity can be acquired through prior infections, maternal passive immunity, or vaccination 28. Moreover, DENV may cross-react with other viruses, thereby potentially triggering the ADE phenomenon. This is evidenced by the fact that specific B cells from individuals infected with DENV have been shown to facilitate ZIKV infection *in vitro* 31.

Fcγ receptors (FcγRs) are integral to the pathogenesis of DENV-ADE, as they possess the capacity to recognize and internalize non-neutralizing antibodies (Nabs) bound to DENV, leading to an increased viral load and potential exacerbation of the disease 3,29,32. It has been reported that BHK cells expressing FcγRIIA exhibit a 10-fold increase in viral titer after DENV infection compared to cells lacking FcγRIIA expression when cultured with serum from patients experiencing secondary infections 29. ADE is associated with persistent inflammation, lymphopenia, and the induction of a cytokine storm, which can lead to severe illness or even death 2,32. It is hypothesized that alterations in cytokine profiles and antiviral response molecules are critical in the pathogenesis of DENV diseases exacerbated by ADE. In the context of FcγRs-mediated DENV-ADE, there is a down-regulation in the expression levels of antiviral type I interferons and interferon-stimulated genes, such as IRF1, NOS2, RIG-I, and MDA-5, while the levels of IL-6 and IL-10 are up-regulated 29. ADE presents a substantial obstacle in the advancement of dengue virus vaccines. In clinical trials of the tetravalent dengue vaccine (CYDTDV), individuals who were initially seronegative exhibited a heightened rate of hospitalization and more severe disease symptoms following subsequent infection with the DENV. This phenomenon may be attributed to the suboptimal efficacy of the vaccine against serotypes 1-3 or the inability of T cells to elicit cell-mediated immunity, as they primarily recognize non-structural proteins that are absent in the chimeric vaccine, thus potentially triggering the ADE effect 2,28,32.

Coronaviruses (CoVs) are a class of enveloped, single-stranded RNA viruses characterized by four genomes encoding structural proteins: the Spike protein (S), the Envelope membrane protein (E), the Membrane protein (M), and the Nucleocapsid protein (N) 33. The transmission of certain highly pathogenic coronaviruses represents a substantial threat to both human and animal health, a risk that is further amplified by the ADE phenomenon. Multiple studies indicates that cats vaccinated against FIPV exhibited more severe illness compared to their unvaccinated counterparts 34,35. This increased severity may be attributed to the enhancement of viral uptake by macrophages, which is mediated by virus-specific antibodies. *In vitro* studies have shown that the ADE of FIPV in macrophages can be augmented by monoclonal antibodies (mAbs) of the IgG2a subclass 36. Studies have indicated that MERS-CoV can productively infect macrophages, with an observed increase of immune cell presence in infected bone marrow cells, leading to heightened chemokines production 32. Furthermore, it has been confirmed that individuals exposed to MERS-CoV who fail to generate neutralizing antibodies face an increased risk of developing severe pulmonary disease upon reinfection 37. Neutralizing antibodies have the capacity to recognize and block the receptor binding domain (RBD) and the heptapeptide repeat 2 (HR2) domains on the S protein of SARS-CoV, which can interfere with the viral attachment to atypical angiotensin-I-converting enzyme 2 (ACE2), and enhance the immune response involving phagocytes, complement system, and NK cells 32.

Antibodies demonstrate a bifurcated impact on coronavirus infections, as evidenced by the occurrence of the ADE phenomenon during SARS-CoV infection. While high concentrations of antiviral serum can effectively neutralize SARS-CoV infection, suboptimal concentrations of neutralizing antibodies may significantly increase SARS-CoV infection and trigger the ADE effects 2,38. Considering the structural similarities between SARS-CoV and SARS-CoV-2, it is imperative to account for the potential cross-reactivity among various coronavirus antibodies in the process of developing vaccines and neutralizing antibody therapies for SARS-CoV-2 2. Research indicates that NAbs isolated from individuals with either acute or convalescent SARS-CoV-2 infections, or those with a prior history of SARS-CoV infection, predominantly target an epitope within the N-terminal domain (NTD) of the S protein, which is located at a site distal to the host cell binding region. This targeting has been shown to enhance ACE2 binding and facilitate SARS-CoV-2 infection via FcγRs mediation 39,40. A reduction in antibody levels may lead to secondary infections, thereby heightening the risk of ADE in children and exacerbating vaccine-induced viral infections 28,41. Additionally, elderly people with prior exposure to various coronavirus antigens are more susceptible to experiencing ADE, whereas younger individuals with limited antigen exposures tend to exhibit more targeted antibody responses to viral neoantigens 42. This variation in immune response may partially explain the heightened immunopathological effects of COVID-19 observed in the elderly population.

ADE transpires through heightened viral uptake and replication and/or intensified immune activation, attributable to the presence of specific sub-neutralizing antibodies 43. Currently, there is a lack of clinical evidence, immunological analyses, or biomarkers capable of differentiating between severe viral infections and immune-enhancing conditions 32. The phenomenon of ADE mediated by antibodies generated in COVID-19 patients has not been documented in clinical trials. However, *in vivo* and* in vitro* investigations, alongside preliminary clinical studies conducted in China, have demonstrated that immunoglobulin responses are generally elevated in patients with severe disease manifestations 38. A dynamic model has been employed to evaluate the potential impact of ADE on SARS-CoV-2, confirming that ADE may expedite the progression of SARS-CoV-2 infection. These findings imply that merely augmenting antibody titers, without a concurrent enhancement of neutralizing capacity may be inadequate for controlling SARS-CoV-2 infection in the presence of ADE 44. Consequently, there is an urgent need for comprehensive studies to elucidate the clinical significance of the relationship between protective immunity against SARS-CoV-2 and the effects of ADE. Moreover, existing research lacks the capacity to accurately forecast the potential development of ADE effect in the context of SARS-CoV-2, especially concerning immune interventions such as vaccination or antibody therapies. The prediction and confirmation of ADE are further complicated by the lack of specific laboratory markers 45. Therefore, the potential for antibodies to contribute to ADE, exacerbating pathogenesis during SARS-CoV-2 infection, vaccination, or through the use of neutralizing antibodies and convalescent plasma therapy (CPT), remains uncertain 46. Ongoing investigations are necessary to ascertain the safety of vaccines and antibody therapies against human coronaviruses 32.

## 2. Available evidence and potential mechanisms of ADE in coronaviruses

### 2.1 Available evidence of ADE reported in coronaviruses

Although limited evidence suggests that SARS-CoV-2 may trigger the release of cytokines and chemokines via ADE. The prevalence of cytokine storms in infections caused by other highly pathogenic human coronaviruses necessitates careful consideration of the potential for ADE to result in aberrant cytokine release by augmenting the antigen presentation of SARS-CoV-2 within cells 47. Experimental investigations have shown that neutralizing antibodies can induce ADE through conformational alterations of S proteins in HEK293T cells 25,42. Furthermore, it has been established that SARS-CoV-2 possesses the capacity to incite cytokine storms by infecting human macrophages and dendritic cells, thereby stimulating the production of inflammatory factors, a mechanism akin to that observed in SARS-CoV 47,48. Given that macrophages exhibiting a pro-inflammatory phenotype are capable of infiltrating the pulmonary system in patients with severe COVID-19, it is plausible to hypothesize that SARS-CoV-2 infection of alveolar macrophages could result in an imbalance of immune responses, ultimately culminating in a cytokine storm 47. A cross-species single-cell RNA sequencing (scRNA-seq) analysis also revealed that cytokine-producing macrophages, in conjunction with pneumocytes, were the primary contributors to viral transcription in both Syrian hamsters and African green monkeys. Furthermore, lung macrophages were identified as playing a pivotal role in severity of COVID-19 mediated by ADE 39,49.

An alternative mechanism of viral infections, ADE, has been identified as being caused by SARS-CoV-2 infection, which is associated with both cellular phagocytosis and endocytosis 27. Immune cell receptor molecules, such as Fc receptors (FcRs), bind to immune complexes formed by viruses and antibodies, facilitating their internalization and potentially enhancing viral entry 50. Given that macrophages and monocytes express the FcRs (FcRIA, FcRIIA, and FcRIIIA), they have emerged as primary mediators of infection-related ADE 50. The study demonstrated that the monoclonal neutralizing antibodies MW01 and MW05 have the capacity to enhance the infection of the SARS-CoV-2 pseudovirus in B cells expressing FcγRIIB *in vitro*. The X-ray crystal structure determination and the S-trimer binding model reveal that MW01 and MW05 can bind to RBD in S-trimer in both the "up" and "down" conformations. Conversely, the neutralizing mAb MW07, which lacks ADE activity, binds exclusively to RBD in an "up" conformation 27. Moreover, a lower dose of convalescent plasma does not facilitate the entry of SARS-CoV-2 into human monocyte-derived macrophages (hMDMs) and monocyte-derived dendritic cells (MDDCs), nor does it influence cytokine expression, or induce cell death. This suggests that ADE resulting from macrophage infection does not occur in convalescent plasma therapy 48. The study utilized a model of cells expressing CD16 or CD89 in conjunction with Delta (B.1.617.2 lineage) and Omicron (B.1.1.529 lineage) variants of SARS-CoV-2 to assess the ADE activity in serum samples from 26 vaccinated individuals, of which 21 samples tested PCR positive for SARS-CoV-2 *in vitro*. The findings revealed no evidence of FcγRIIIa- and Fcα receptor (FcαR) I-dependent ADE in SARS-CoV-2 infection following prior immunization 51. This suggests that neutralizing mAbs do not facilitate the productive infection of SARS-CoV-2 in immune cells *in vitro*. Current clinical evidence does not conclusively indicate that existing COVID-19 therapies result in an enhanced immune response consistent with ADE 43. Consequently, further research is required to elucidate the potential ADE effects associated with SARS-CoV-2.

Recent animal studies have predominantly demonstrated that the authorized SARS-CoV-2 vaccines do not induce ADE *in vivo*. Certain inactivated vaccine candidates have been shown to elicit high levels of neutralizing antibodies across multiple animal models, indicating robust neutralizing capabilities 52. Notably, the purified inactivated vaccine candidate PiCoVacc has been observed to induce antibodies capable of neutralizing 10 representative SARS-CoV-2 strains. Furthermore, administration of 3 μg or 6 μg doses provided partial or complete protection in macaques, with no evidence of ADE observed 53. Preclinical data have demonstrated that a 2 μg dose inactivated vaccine BBIBP-CorV effectively protects rhesus macaques from intratracheal challenge with SARS-CoV-2 42,54. Apart from inactivated vaccines, several other vaccine candidates have demonstrated protective effects against SARS-CoV-2 challenges in animal models without significant ADE. These vaccines include the adenovirus-based vaccine ChAdOx1 nCoV-19 55, the REVC-128 nanoparticle vaccine candidate 56, the ARCoV mRNA vaccine encapsulated in lipid nanoparticles encoding the SARS-CoV-2 RBD 57, and peptide vaccine candidates optimized to stimulate both T cells and B cells epitope-region enhancement 58.

Research conducted by Dapeng Li *et al.* demonstrated that antibodies targeting RBD and NTD in human convalescent sera can simultaneously mediate both neutralization and enhancement of infections *in vitro*. Nevertheless, these antibodies still exhibited inhibitory effects on viral replication in mouse and macaque models 39. Furthermore, CT-P59, a human-derived mAb targeting RBD, has been proved effective in neutralizing SARS-CoV-2 isolates including the D614G variant, across various animal models 59. Likewise, the mAb IgG1 ab1 also demonstrated significant prophylactic and therapeutic efficacy in hamster models infected with SARS-CoV-2, with no observed ADE phenomenon associated with these antibodies 60. A prophylactic intranasal antibody B8-dIgA, effectively reduced the viral loads of SARS-CoV-2 in lungs, however, it unexpectedly resulted in elevated viral titers and additional damage in nasal turbinates. This RBD-specific dimeric IgA was exploited by SARS-CoV-2 to mediate viral transmission between cells, suggesting a previously unreported mechanism for antibody-mediated nasal infection and injury of SARS-CoV-2 61.

The discrepancy in protective efficacy observed between *in vitro* and *in vivo* setting may be attributed to the virolytic activity mediated by IgG antibody in conjunction with complement involvement. For instance, mAb belonged to IgG3 subclass exhibits both neutralizing activity and enhanced complement-mediated lysis (CoML) activity against various rabies virus strains, including CVS, ERA, HEP-Flury, and Nishigahara 62. The activity of CoML against HIV-1 has also been evidenced through the interaction of a murine mAb, NM-01, with human or rabbit complement, resulting in a significant reduction in viral infectivity 63. While it is commonly assumed that heterotypic antibodies to DENV may exacerbate the secondary infections, recent studies suggest that certain IgG subclasses confer protection against dengue hemorrhagic fever/dengue shock syndrome (DHF/DSS), and exhibit cross-reactive protection against ZIKV virions, potentially inducing virolysis as a main feature 64.

Another factor contributing to this difference may be attributed to antibody-dependent cellular cytotoxicity (ADCC), a Fc-mediated effector function that plays a crucial role in defense against viral infections, beyond mere neutralization 65. ADCC is typically induced by NK cells, which, both *in vitro* and *in vivo*, exhibit significant protective activities against SARS-CoV-2 through ADCC directly or cytokine secretion indirectly 66,67. It is shown that ADCC is more pronounced in patients with mild symptoms. This is accompanied by a decreased expression level of activated receptor NKG2D in peripheral blood NK cells, and an increased level of cell-free NKG2D ligands (NKG2DL) in the plasma, potentially inhibiting the ADCC function of NK cells from healthy donors 68. Conversely, M-specific ADCC activity is high in patients with severe disease 67, a variation that may be attributed to the antibody specificity. The S-specific ADCC activity is higher in symptomatic individuals infected with the wild-type (WT) SARS-CoV-2, whereas no significant difference in N-specific ADCC activity levels is detected 69. In general, Omicron infections exhibit a decline in total ADCC activities compared with WT SARS-CoV-2 infections 69,70, ADCC against S1, RBD, NTD are likely to allow immune evasion by the Omicron BA.1 variant, which could be resisted by ADCC against S2 subunit 71. Given that most SARS-CoV-2 vaccines predominantly induce antibody against S protein, a novel vaccine strategy of incorporating both S1 and S2 antibodies has been proposed 65. Numerous *in vivo* experiments have proved that protections mediated by ADCC are strongly cross-reactive against various coronaviruses, including Omicron variants 72, and possess a higher activity in patients experiencing reinfection 73,74.

Although the occurrence of ADE *in vivo* requires further validation, both prophylactic and therapeutic strategies continue to pose potential risks. Previous studies have demonstrated that the SARS-CoV vaccines can induce ADE across various animal models, including mice, hamsters, ferrets, and macaques 3. Consequently, the manifestation of ADE effects in SARS-CoV has been substantiated through animal experiments. Infection with SARS-CoV in vaccinated macaques resulted in fatal acute lung injury. The cytokine profiles in these macaques closely resembled those observed in macrophages exposed to SARS-CoV and serum from patients who succumbed to the infection 3,75. Matveev A *et al.* was first to report on the limited neutralizing capacity against SARS-CoV-2 of mAb RS2 despite its high affinity. This characteristic was associated with an increased viral load in the respiratory tracts of animal models, thereby exacerbating SARS-CoV-2 infection. In the serum of patients with severe COVID-19, there is evidence of competitive binding for antibodies targeting epitopes recognized by ADE-induced mAb, further supporting the hypothesis that certain antibodies may enhance SARS-CoV-2 infection in human 46. Additionally, some certain S protein-specific IgG antibodies cause severe acute diffuse alveolar damage by disrupting the immune response in multiple animal models. These IgG antibodies, thought to facilitate infections, promote SARS-CoV entry into human immune cells via the conventional FcγR II and ACE2 pathway, warranting further investigations. Given the structural similarities between SARS-CoV-2 and SARS-CoV, the ADE phenomenon in the study of SARS-CoV-2 also merits close examination.

The question of whether coronaviruses can clinically induce ADE effect should be discussed individually for each virus. In clinical samples of SARS-CoV, evidence indicates that the virus can infect immune cells lacking the ACE2 receptor 76,77. Certain antibodies have been observed to facilitate SARS-CoV entry into human macrophages and B cells through the interaction of FcγRs with viral S protein *in vitro* 17,18. In instances of MERS-CoV infection, neutralizing antibodies have been observed to directly bind to the RBD of the S protein via an Fc-mediated targeting effect 3. Thomas S *et al.* conducted ADE assay utilizing serum from MERS-CoV recovery patients and SARS-CoV-2 vaccine recipients, employing BHK cells expressing FcgRIIa. Samples from patients infected with MERS-CoV exhibited SARS-CoV-2-targeting ADE, which was significantly correlated with low levels of neutralizing antibodies. Among the seven patients who received the SARS-CoV-2 vaccination following MERS-CoV infection, only one exhibited ADE induced by SARS-CoV-2 pseudoviruses (PVs) 78. Clinically, it has been observed that the frequency and peak levels of antibodies in patients with severe SARS-CoV-2 infection are higher than those in patients with mild infection. However, there is insufficient evidence to conclusively attribute this phenomenon to ADE 79. Clinical findings regarding this administration of neutralizing antibodies for SARS-CoV-2 suggest an absence of ADE 55,80,81. Overall, there is presently no clinical evidence indicating the occurrence of SARS-CoV-2-related ADE *in vivo*. Consequently, further comprehensive investigations into ADE are necessary, as they will provide valuable insights for the development of future vaccines and antibody-based therapeutic strategies.

### 2.2 Potential mechanisms of ADE

**Figure 1:**
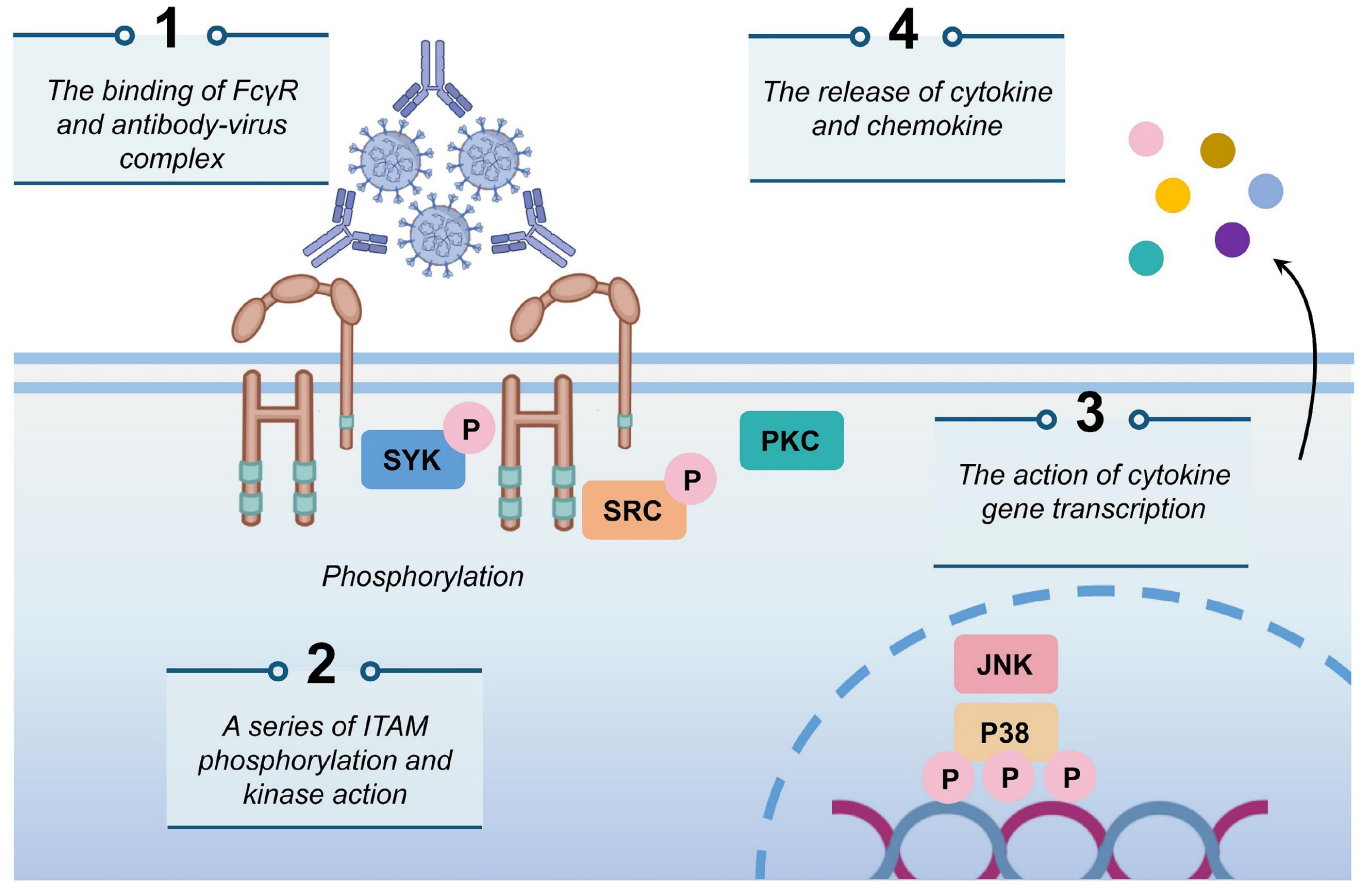
** The mechanism of ADE mediated by FcγR.** The antibody-virus complex binds to FcγR on the cell surface, initiating a cascade of events characterized by the phosphorylation of immunoreceptor tyrosine-based activation motifs (ITAMs) by Syk and PI3K. This activation triggers the FcγR-mediated phagocytosis signaling pathway, which subsequently facilitates viral infections and the release of cytokines and chemokines.

**Figure 2:**
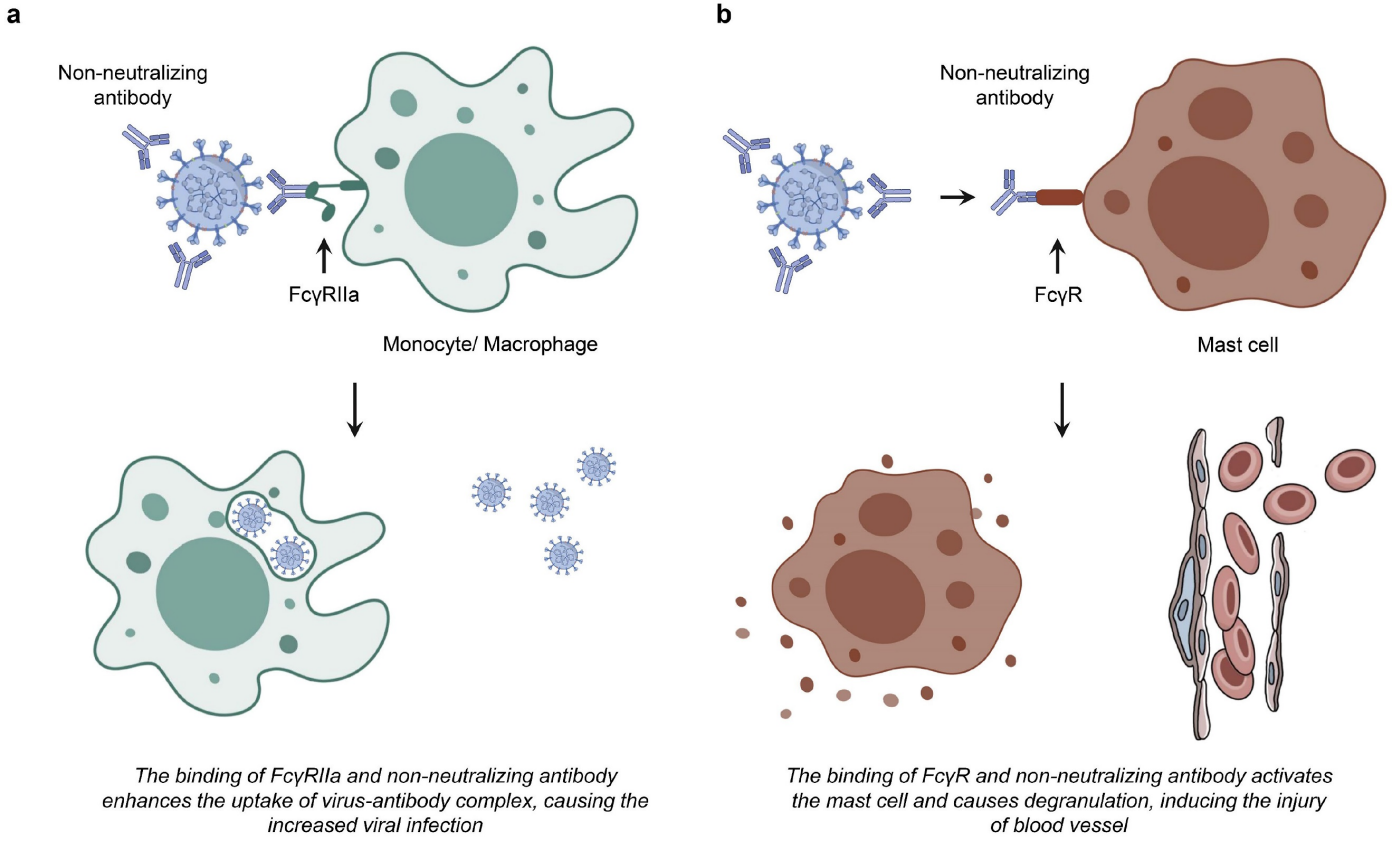
** Two hypotheses of FcR-expressing cells infected by coronavirus. a.** The interaction between non-neutralizing antibodies and FcγRIIa results in the formation of a virus-antibody complex, potentially facilitating increased viral uptake and subsequent infection in monocytes and macrophages. **b.** The binding of non-neutralizing antibody to FcR induces the activation and degranulation of mast cells.

To more effectively mitigate the potential risk of ADE associated with SARS-CoV-2 infections, it is imperative to deepen our understanding and investigation of its mechanisms. The mechanisms contributing to the ADE effect are complex, among which, the binding mechanism involving FcγRs has been extensively documented. In this process, specific antibodies attach to the virus, forming virus-antibody complexes that facilitate the direct transport of the virion to macrophages. Antibodies transport the virus to the cell surface for further binding to the FcγR, leading to the phosphorylation of Syk and PI3K and the activation of the FcγR-mediated phagocytosis signaling pathway, thereby promoting viral entry (Fig. 1) 34,43,82,83. Previous studies on DENV have demonstrated that antibodies of different serotypes mediate viral entry into cells via the FcγR on phagocytic monocytes, consequently, increasing viral loads *in vivo* 84. In the context of coronavirus research, Jaume M *et al.* identified that antibody-mediated infection operates via FcRII pathway, as opposed to the conventional ACE2-dependent mechanism 18, which is similarly implicated in SARS-CoV-2 infections 85. FcγR predominantly expressed on immune cells such as macrophages, B cells, and natural killer cells, serve as a receptor for the Fc region in antibodies 83,86. The antibody-FcR complex can functionally mimic the viral receptor, thereby enabling the virus to expand its host cell tropism through the FcR-mediated pathway (Fig. 2a) 25. To date, both mAbs and polyclonal serum have been shown to mediate ADE phenomenon *in vitro* using various FcRs-expressing cells, including K562 and U937 cell lines, as well as primary human monocytes, macrophages, and dendritic cells 87. Furthermore, studies employing Vero E6 cells expressing FcγIIa have confirmed that ADE antibodies against Ebola virus (EBOV) ZGP12/1.1 88 enhance the transmissibility of pseudotyped VSV bearing the EBOV glycoprotein (GP) (VSV-EBOV) 86. In ADE-induced SARS-CoV infection, ST486 cells were engineered to express different types of FcγRII ectodomains and transmembrane domains. The study demonstrated that while IgG-SARS-CoVpp immune complexes are capable of binding to these cells, this binding alone is insufficient to facilitate viral entry. This finding indicates that the intracellular signaling motif of FcγR, rather than the IgG-binding motif, serves as the key molecular determinant for SARS-CoVpp infection 17.

Some specific mAbs against the SARS-CoV-2 S protein have been observed to facilitate viral infection in human B lymphocytes and chronic granular leukemia cell lines through an FcγRs-mediated pathway *in vitro* 50,89,90. FcγRII (CD32) is recognized for its affinity to IgG and can be activated by virus-antibody immune complexes 83. The infection of CD32^+^ cells infection is considered a critical factor in the progression of COVID-19 from mild to severe stage 91,92. The prevailing hypothesis regarding the ADE mechanism in SARS-CoV-2 infection posits that Nabs bind to the virus but lack neutralizing capacity, and the virus-Nab complex interacts with immune cells through FcRs. This interaction is believed to lead to a significant increase in the production of pro-inflammatory cytokines while concurrently inhibiting the secretion of anti-inflammatory cytokines 93. As supplementary, the emergence of the multi-system inflammatory syndrome in children (MIS-C) during the COVID-19 epidemic indicates a potential mechanism of the ADE phenomenon. This mechanism involves IgG and IgE antibodies against SARS-CoV-2 binding to FcRs on mast cells, resulting in their activation and degranulation, which subsequently elevates histamine levels *in vivo* 83,94. Elevated histamine levels are anticipated to impede myocardial capillary blood flow as a result of pericellular contraction, subsequently increasing the risk of coronary artery aneurysm associated with cardiac pathology and contributing to increased blood pressure due to hypoxia-induced cell apoptosis (Fig. 2b) 94. Cardiac injury is frequently observed as a clinical symptom among hospitalized patients with COVID-19 and is correlated with high mortality rate 95. Nonetheless, the clinical implications of ADE in the context of SARS-CoV-2 infection remain a subject to debate, as FcγRs are exclusively expressed on immune cells and do not constitute the primary targets of SARS-CoV-2 86.

Antibodies acquired through different mechanisms can lead to ADE, exemplified by coronavirus antibodies generated from repeated infections with non-pathogenic coronaviruses. Coronaviruses share multiple cross-reactive epitopes, and upon reinfection with coronaviruses, non-neutralizing cross-reactive antibodies are initially produced. These antibodies facilitate viral entry into mononuclear and macrophage cells via FcR mediation 42,96. Moreover, maternally transferred antibodies (matAbs) are also thought to mediate the production of ADE through FcγRII. Infants with MIS-C associated with COVID-19 may acquire antibodies that bind to FcRs on mast cells, exacerbating disease progression 47. FcRs-mediated ADE enhances viral entry by increasing the binding efficiency of the virus-antibody complex to FcRs, as vaccine-induced antibodies fail to neutralize the virus owing to the insufficient concentration, affinity, or specificity 34. ADE is more probable at low antibody concentrations, and the risk of ADE escalates when antibody concentrations diminish post-vaccination 93,97. In case of SARS-CoV, it has been established that high concentrations of serum can neutralize the virus; however, infections may be exacerbated when the serum is highly diluted, a process mediated by anti-S protein antibodies present in the serum 16,85,98.

**Figure 3:**
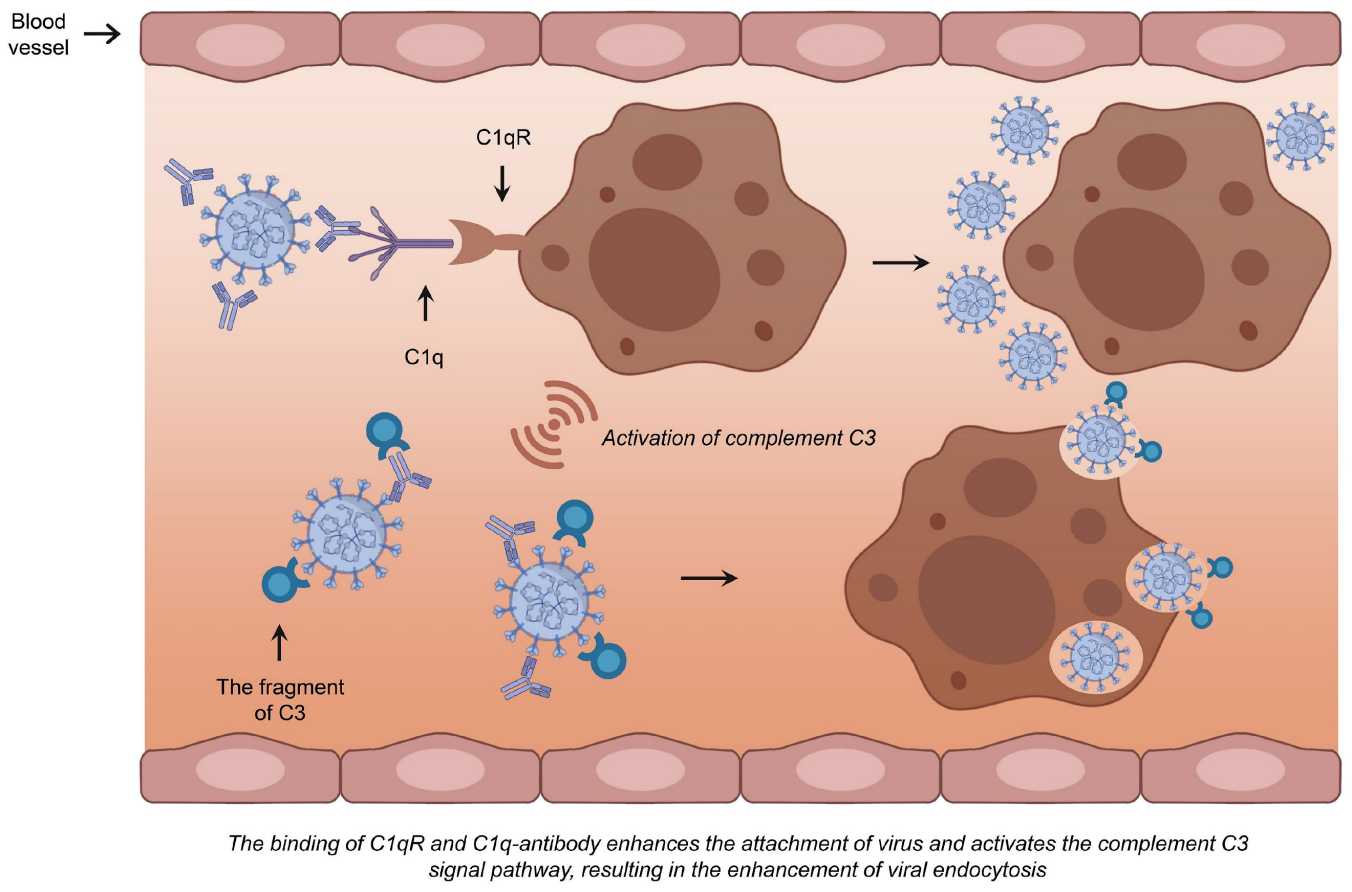
** The mechanism of ADE mediated by C1q.** The binding of C1q to the antibody-virus complex may subsequently interact with C1qR, facilitating increased viral aggregation on the cell surface. This interaction initiates the intracellular signaling pathways and activates C3, which can mediate subsequent membrane fusion and endocytosis, and consequently enhance viral infection by linking to the antibody-virus complex.

The C1q complement pathway is regarded as the second most significant contributor to ADE. C1q, a crucial component of C1 complex in plasma, has the capacity to bind to virus-antibody complex. Upon binding to the C1q receptor (C1qR), C1q facilitates the aggregation of more viral particles on the cell surface 86. This interaction subsequently triggers the intracellular signaling pathway, leading to the activation of C3 following the binding of the C1q-complex to the C1q receptor. By covalently attaching to either the bound antibodies or the viral particles surface, C3 fragments facilitate the binding of the virus to its receptor. This process promotes the subsequent fusion and endocytosis between the viral envelope and the cell membrane, thereby enhancing viral infection (Fig. 3) 83. Several studies have suggested that the mechanism of ADE primarily involves the FcγR. Since FcγRs expression is restricted to immune cells, including macrophages, B cells, and natural killer cells, which are not the main targets of SARS-CoV-2, it is plausible that the C1q receptor mechanism offers a more persuasive explanation for ADE-mediated SARS-CoV-2 infection of respiratory epithelial cells 86.

Consider the example of FIPV, where numerous viral and cellular molecules participate in the ADE process. FIPV, a member of the feline coronaviruses (FCoV), is recognized as utilizing feline aminopeptidase NAPN (fAPN) as a viral receptor 99. Recent studies have validated the ADE phenomenon mediated by anti-S protein mAbs of FIPV *in vitro*, as well as through recombinant vaccinia virus *in vivo* 11,82,100-102. Multiple studies have demonstrated that viral production is significantly elevated in feline alveolar macrophages when FIPV is mixed with mAbs. This increase can be mitigated by pre-treating IgG with protein A 12,103,104. Corapi WV *et al.* identified that the majority of mAbs belong to the IgG2a subclass capable of inducing ADE 11. However, in the human mononuclear cell line U937, which lacks the FIPV receptor, both IgG2a and IgG1 subclasses can induce viral production when the cell is inoculated with FIPV 104. The results indicate that ADE in FIPV infection is mediated by the Fc region of IgG2a. This is corroborated by another finding demonstrating that FcγR I and II, expressed on the surface of U937 cells can bind to IgG2a and IgG1, respectively 104. Additionally, ADE may also result from the cross-reactivity of antibodies against type I FIPV strains KU-2 or UCD-1 with type II FIPV 79-1146 strain, as well as from reinfection with the 79-1146 strain 12. Conversely, the study by Takano T *et al.* found that FIPV-infected macrophages induced by ADE exhibited an increased mRNA expression level of fAPN. This up-regulation contributes to heightened viral sensitivity and earlier onset of FIP symptoms 103.

## 3. Discussion on ADE phenomenon of different therapies

### 3.1 Vaccination

The emergence of SARS-CoV-2 and its variants has significantly impacted on global health, underscoring the necessity for the development of effective and safe vaccines to mitigate the incidence of severe cases. In contrast to SARS-CoV-2, the two highly lethal human coronaviruses, SARS-CoV and MERS-CoV, exhibited mortality rates ranging from 9% to 40%, respectively. However, neither virus reached pandemic proportions, and substantial advancements in vaccine development have been limited 42. Currently, several subunit vaccines based on the S-glycoprotein have been developed for MERS-CoV and SARS-CoV 105, although there are limited reports regarding their safety. An exacerbation of respiratory disease (ERD) has been documented in mouse and nonhuman primate (NHP) models of pneumonia following viral exposure 106,107. Vaccination of hDPP4-mice with whole inactivated virus of MERS-CoV elicited an eosinophil-containing pulmonary immunopathology, despite its protection against viral infection 107. The antibodies induced by S-glycoprotein peptide S597-603 enhanced SARS-CoV infection both *in vitro* and in rhesus macaques 106. Fortunately, this phenomenon has not been observed in clinical trials involving SARS-CoV-2, the extensive and accelerated deployment of SARS-CoV-2 vaccines has become a success worldwide which is lacking of significant safety considerations.

Recent studies indicated that the inactivated SARS-CoV-2 virus vaccine, PiCoVacc, and the human adenovirus vaccine, ChAdOx1 nCoV-19, both confer protection against SARS-CoV-2. Furthermore, no ADE phenomenon was observed in preclinical studies involving rhesus monkeys 42,53,105. Besides, many vaccines, including the inactivated vaccine BBIBP-CorV 108, the mRNA vaccine BNT162b2 109,110, and the protein subunit vaccine ZF2001 111,112, have successfully completed phase III clinical trials demonstrating their safety and efficacy without significant adverse side effects and ADE activity. Current SARS-CoV-2 vaccines against the Omicron variant have been shown to significantly boost virus-neutralizing antibody in the serum against emerging subvariants such as HV.1, HK.3, JD.1.1, and JN.1 113. Nevertheless, the emergence of new Omicron variants XBB1.5 and BQ.1.18 has underscored the necessity for more efficacious vaccine strategies 114. Although the XBB1.5 vaccine provided considerate protection against SARS-CoV-2 infection during the initial 3 months post-vaccination, studies indicated that its vaccine effectiveness (VE) across all age groups was below 50%, with even lower efficacy observed against the JN.1 variant 115.

Preclinical investigations into coronavirus vaccines have indicated that vaccine-associated enhanced disease (VAED) may result from a sub-neutralizing state of antibodies generated against various structural proteins or a modified S protein. Phenomena such as ADE, VAED, or antibody-enhancing disease (AED) have been observed in animal models of FIPV, MERS-CoV, and SARS-CoV. Although these phenomena have not been observed in clinical cases, they must still be taken into account in vaccine development and safety research due to the potential concurrence of VAED and other microbial infections, which may exhibit a propensity for ADE and/or AED 116. Moreover, research indicates that ADE might occur in individuals who have received mRNA or viral vector vaccines based on the wild-type strain S protein and are subsequently exposed to the Delta variant of SARS-CoV-2 117. The NTDs-targeted neutralizing antibody 4A8, which is detected in COVID-19 patients, exhibits a marked reduction in efficacy against the NTD of Delta variants, indicating a substantial decrease in neutralizing activity against these variants 118,119. Similarly, recent studies have demonstrated that the anti-NTD antibody has also lost its binding capacity with the emergence of the new Omicron variant 120,121. The mutations of Omicron variant have been linked to antibody evasion, resulting in a reduced binding affinity of antibodies and consequently influencing their neutralizing efficacy 122,123. The effectiveness of antibodies can differ according to the specific strain, suggesting that SARS-CoV-2 vaccines based on the S protein of the original strain may become less effective in preventing infections as new variants continuously emerging.

### 3.2 Neutralizing antibodies

This method has been proven effective for isolating and developing therapeutic antibodies from the memory B cells of convalescent patients 39,124. These antibodies have demonstrated protective effects in animal models against coronavirus infections, including SARS-CoV, primarily by inhibiting the interaction between the RBD on the S protein and the ACE2, thereby attenuating or abolishing SARS-CoV-2 infection 125,126. Chan C *et al.* 125 reported that the IgG1 antibody SC31, which targets S protein RBD and is isolated from patients in the early convalescence stage can induce an antiviral response driven by Fc-mediated effectors. This antibody effectively inhibits SARS-CoV-2 infection by blocking the binding of the S protein to the human ACE2 receptor, without causing ADE. To date, several potential therapeutic neutralizing antibodies against SARS-CoV-2 have been identified, and their prophylactic and therapeutic effects have been demonstrated in mouse, hamster, and NHP models.

However, Lai GC *et al.* 126 reported a mouse-specific antibody targeting the SARS-CoV-2 RBD that was ineffective in neutralizing the Gamma variant with mutation of residue E484K in the S protein. Concurrently, the result from the plaque reduction assay indicated that a dose-dependent increase in viral infectivity with the escalating concentrations of 1Ba-3H. Subsequent research has been undertaken to verify that 1Ba-3H-mediated ADE may enhance infection at a low titer of the Gamma variant 127. Given that the molecular mechanisms underlying ADE mediated by remain clear, it is imperative to identify its binding epitope on the S protein, in order to better understand its potential neutralizing activity or the ADE mechanisms that might facilitate viral entry 127.

Some ADE phenomena observed *in vitro* may not manifest *in vivo*. In the study conducted by Li D *et al.* 39, neutralizing antibodies targeting the RBD and the NTD of the SARS-CoV-2 S protein were isolated respectively. The RBD antibodies demonstrated FcγRs-mediated infection-enhancing activity *in vitro*, while the five non-neutralizing NTD antibodies were capable of mediating FcγRs-independent infection enhancement *in vitro*. However, all of these *in vitro* infection-enhancing antibodies have exhibited protections from SARS-CoV-2 challenge in macaque and mouse models. The precise infection mechanism remains unclear; however, elevated levels of NTD-targeted antibodies have been observed in severe patients, albeit the presence of these potential infection-enhancing antibodies in uninfected donors. These antibodies have been reported to enhance the infectivity of SARS-CoV-2 by promoting stronger interaction between the S protein and the ACE2 128. *In vitro* studies have demonstrated that infection-enhancing antibodies can inhibit SARS-CoV-2 replication or prevent pulmonary infections in both monkey and mouse models. Further experiment revealed that Fc effector functions facilitate the neutralization of antibodies C104, C002, and C110, thereby conferring protection in mice 129. Some researchers hypothesize that these *in vitro* enhanced antibodies may possess the capacity to suppress SARS-CoV-2 replication *in vivo* through FcRs-mediated effector functions 41,129.

Studies have indicated that ADE effects may be mediated by antibodies targeting the nucleocapsid protein. Yasui F *et al.* have demonstrated that the N protein can up-regulate the expression of cytokines IFN-γ, IL-2, IL-4, and IL-5 and pro-inflammatory cytokines IL-6 and TNF-α, and concurrently down-regulating the expression of anti-inflammatory cytokines IL-10 and TGF-β in SARS-CoV-infected BALB/c mice. This modulation of cytokine expression results in the infiltration of neutrophils, eosinophils, and lymphocytes into the lung tissue, accompanied by thickening of the alveolar epithelium, thereby contributing to the pathogenesis of severe pneumonia 130. Similarly, the SARS-CoV-2 N protein not only activates IL-6 and IL-1β, but also induces a hyperinflammatory response by promoting the activation of the NLRP3 inflammasome in cultured macrophages and dendritic cells, as well as in C57BL/6 mice. This activation exacerbates pulmonary injury and accelerates mortality in mouse models of sepsis and acute inflammation 131. Clinical data from the early stages of the COVID-19 pandemic have demonstrated significant elevations of IL-6 levels in serum from patients with severe symptoms 132,133, which correlates with adverse clinical outcomes and high mortality rate among hospitalized patients 134. Notably, the excessive recruitment of IL-6-secreting monocytes to the infected lungs is associated with severe symptoms of SARS-CoV-2 infection 135. It has been observed that N protein can progressively enhance IL-6 production in myeloid cell K-ML2 132, as well as in monocyte-derived macrophages (MDM) 136. Moreover, the addition of anti-N antibody further enhanced IL-6 production in K-ML2 cells, indicating that the anti-N antibody may contribute to the cytokine storm associated with COVID-19 136. Nevertheless, it is important to note that only peripheral blood MDM and induced pluripotent stem (iPS) cell-derived myeloid cells but not models of alveolar macrophages were utilized in this study, which may not accurately mimic the immune response in pulmonary environment 136. Nakayama EE *et al.* have demonstrated that the N proteins of both the Delta and Omicron BA.1 variant significantly suppress IL-6 production in myeloid cells 137. This finding aligns with the observation that the Omicron variant exhibits reduced pathogenicity in hamster models 138. Additionally, the concentration of anti-N IgG in serum has been demonstrated to correlate with clinical outcomes. At the time of admission, 55% of patients were positive for the anti-N antibody in serum, suggesting that the anti-N IgG titer could serve as a prognostic factor for SARS-CoV-2 infection 134. And these clinical studies are constrained by the small sample size and the multiple variables, further investigation is required to elucidate the pathological mechanisms linking anti-N antibodies to severe symptoms of COVID-19. Even so, the cytokine storm induced by anti-N antibodies has been hypothesized as a potential explanation for the mechanism of ADE.

Over 100 therapeutic mAbs that leverage the potent FcγRs-mediated effector systems have been approved for the treatment of various diseases, whose receptors can interact with antigen complex IgG ligands to activate and modulate the robust inflammatory functional network that safeguards the host 139. Consequently, several highly efficacious neutralizing mAbs against the SARS-CoV-2 S protein RBD have also been identified and assessed in clinical trials 140. A wild-type IgG1 antibody, MW05 (MW05/IgG1), has been shown to enhance the infection of SARS-CoV-2 pseudovirus in Raji cells through the interaction of Fc with high expression levels of FcγRIIB 141. ELISA assays identified the key amino acid residues involved in the binding interaction with MW05 as E484 and F490. This distinct recognition sites, compared to other mAbs, are likely responsible for MW05-induced ADE 141. It is noteworthy that MW05 and MW06 share an identical Fc region while possessing distinct Fab regions. This structural variance results in differential recognition conformations and binding sites on the S protein for the two antibodies, which may involve in the ADE mechanism mediated by mAbs^140^.

Kim C *et al.* 59 identified an antibody CT-P59, which exhibits a completely different binding site from other RBD mAbs. This antibody effectively neutralizes various SARS-CoV-2 isolates, including the D416G variant without inducing ADE. Therapeutic effects evaluation of CT-P59 across all three animal models, including ferrets, hamsters, and rhesus monkeys, demonstrated a significant reduction in viral titers. Notably, the ferret model exhibited a marked decrease in viral loads two days post-infection when compared to treatment with Remdesivir. Winkler ES *et al.* 142 highlighted that mAbs exhibit superior efficacy in alleviating lung infections and diseases compared to conventional therapeutic drugs. Furthermore, the administration of mAbs demonstrating a strong ADE effect *in vitro* did not lead to similar effects *in vivo*. This observation can be attributed to two primary factors: firstly, SARS-CoV-2 is unable to replicate effectively in macrophages, which are the main targets of Fc-mediated ADE antibodies; secondly, the Fc-mediated effector function plays a protective role, as it has been observed that Fc effectors contribute to the preservation of the activity of SARS-CoV-2 neutralizing antibodies *in vivo* 39. Meanwhile, when the concentration of antibodies in the body is elevated, certain antibodies targeting the S protein cannot neutralize the virus. Instead, they can induce conformation changes in the S protein, thereby enhancing its binding affinity to ACE2 receptors. However, this does not enhance viral infection with high level neutralizing antibodies presenting 143.

### 3.3 Convalescent plasma

Convalescent plasma therapy has been identified as an effective treatment modality for COVID-19. As a form of passive immunotherapy, CPT involves the transfusion of plasma from individuals who have fully recovered from the infection into recipients, to achieve therapeutic purpose 144. Concerning the potential risk of ADE associated with convalescent plasma therapy, it is no longer the priority of doctors to cure COVID-19 patients due to the complex compositions in plasma. Nevertheless, in the early stage of virus outbreaks, this therapeutic approach is employed to aid patients with severe manifestations of the disease 145. The potential occurrence of ADE is of significant concern, attributed to the presence of neutralizing, non-neutralizing, or sub-neutralizing antibodies in convalescent plasma 146. Numerous serological studies on COVID-19 convalescent patients have identified a series of polyclonal lineage specific or cross-reactive antibodies, which can elicit diverse effector functions against various viral variants. Coutant F *et al.* Conducted a study to assess the neutralizing activity of mAbs derived from convalescent patients against the SARS-CoV-2 D614G, Delta, and Omicron (BA.1) variants, this finding revealed that 38 mAbs increased the infectivity of both the Delta and Omicron variants. Additionally, two mAbs demonstrated a neutralizing effect against the Omicron variant while simultaneously enhancing the cytopathic effect of the Delta variant *in vitro* 147. Clark NM *et al.* 148 conducted a study utilizing cells expressing ACE2 viral receptors, FcαR, and FcγRIIA, both individually and in combination, to assess the neutralizing effects of anti-S protein IgG, IgA, and IgM with adapted retroviral-pseudotypes. The findings demonstrated that the neutralization titer of the convalescent plasma was predominantly influenced by IgG concentration, exhibit a positive correlation. Furthermore, ADE mediated by IgA and IgM was not detected in the convalescent plasma. This suggests the absence of ADE induced by S protein retroviral-pseudotype particles and antibodies in the convalescent plasma, even in the presence of FcαR and FcγRIIA 148. Diorio C *et al.* conducted an investigation into donor antibody levels and recipient antibody responses before and after CP input, demonstrating that CP input neither induces ADE nor suppresses the endogenous antibody response 149. The occurrence of ADE involves FcγR, and although hMDMs express FcγR, COVID-19 convalescent plasma does not enhance infection in hMDMs, nor does it activate the innate immune response 150. Furthermore, Wu J *et al.* identified a clonal antibody, XG005, derived from convalescent patients, which demonstrated effective and extensive neutralizing activity against various SARS-CoV-2 variants, with particular efficacy against the Omicron sub-lineages. A structural analysis was conducted to reveal the pivotal role of somatic mutations which endows XG005 with greater neutralizing potency and breadth, along with higher antibody product quality and lower ADE effect 151.

Nevertheless, some studies have reached the opposite conclusion. For instance, Shen XR *et al.* demonstrated that SARS-CoV-2 convalescent serum can augment the infection of primary B cells, macrophages, and monocytes that express varying levels of FcγRs 152. Consequently, prior to the clinical application of convalescent plasma therapy, it is imperative to assess the ADE phenomenon of convalescent plasma using cell lines that express FcγRs receptors or complement receptors, or even ACE2 receptors 153.

## 4. Potential strategies for inhibiting ADE in viral infections

ADE can be categorized into two different types of viral infections: enhanced infection and enhanced immune activation. Enhanced infection is primarily mediated by Fc-FcγR interactions. Consequently, blocking these Fc-FcγR interactions through Fc mutations represents a viable strategy to mitigate potential ADE *in vivo* 27,154. The LALA mutation, which mutates Leu234Ala and Leu235Ala within the Fc region, is a prevalent strategy to decrease the affinity of the Fc region for FcγR, thereby avoiding ADE 38. Empirical evidence has demonstrated that the LALA variant effectively abrogates ADE in DENV and ZIKV infections 155. Furthermore, the application of the LALA mutation to the Fc region of neutralizing antibodies against SARS-CoV-2 is under clinical investigations, aiming to diminish interactions with FcRs and eliminate ADE effects 59,140,156. Wang YT *et al.* 155 reported a development of three mAbs: 1741-LALA, S1D2-hIgG1, and its variant S1D2-LALA generated through LALA mutation. These antibodies demonstrated broad neutralizing activity against various of SARS-CoV-2 mutant strains. In the lungs of SARS-CoV-2-infected mice, the Fc effector function was observed to decrease the viral load by 6-14-fold 142. However, no significant difference in virus-neutralizing capacity was noted between the LALA variant and the wild-type antibody 155. Furthermore, neither SARS-CoV-2 mAbs nor convalescent plasma facilitated infection by the SARS-CoV-2 D614 strain in human monocyte-derived macrophages. The mAb MW05 was shown to completely eliminate the ADE effect 141,157. To prevent the interaction between the Fc region and FcRs, both Fc engineering and the use of IgG4, which possesses a low affinity for C1q and FcγRs while retaining its neutralization effect against the virus, can be employed 157,158. Certain antibodies, such as P4A1, exhibit strong binding affinity to the S protein of SARS-CoV-2. Targeted modifications of these antibodies can significantly mitigate the potential risk of ADE 159. Given that the interaction between the Fc region and FcγR may mediate ADE, employing heterologous polyclonal antibodies that do not interact with human FcRs presents a promising strategy to circumvent this issue. Studies have shown that the modified porcine GH-pAb Fc region is incapable of engaging with human FcRs and recruiting human effector, thereby providing a viable means to prevent ADE 160. To mitigate ADE mediated by FcγRs, it has been proposed that intravenous immunoglobulin (IVIG) may competitively bind to FcγR, thereby reducing the incidence of ADE 156. Additionally, a series of peptides have been engineered to protect ACE2 or S protein neutralizing activity, effectively inhibiting the entry of pseudotyped SARS-CoV-2 virus into HEK293T/hACE2 cells, either independently or in combination 161. Furthermore, Wang S *et al.* 27 demonstrated that FcγⅡRB can mediate ADE of SARS-CoV-2 *in vitro*. However, the presence of other FcγRs did not result in ADE, suggesting that the expression of these other FcγRs on cells may inhibit ADE by competing with FcγⅡRB.

ADE can be resulted from issues related to either the quality or quantity of antibodies, especially the presence of non-neutralizing antibodies or low concentrations of sub-neutralizing antibodies. Ideally, high antibody titers ought to neutralize the virus by inhibiting the binding of the viral protein to the target receptor, otherwise insufficient antibody levels failed to exert a neutralizing effect may facilitate ADE 116. Therefore, inducing antibodies with higher titers, as well as enhancing the specificity of antibodies to more effectively target antigens can mitigate the risk of ADE 44. Within one year post-vaccination, there may be an elevation in non-neutralizing or sub-neutralizing antibody levels as a result of a decline in neutralizing antibody levels. This occurrence could potentially enhance viral infection or lead to pulmonary immunopathology, thereby posing a challenge to the long-term safety of vaccines 162. Consequently, the ADE effect is a critical consideration in vaccine design. It necessitates the careful selection of target antigens, the minimization of non-neutralizing antibody-inducing regions, and prioritization of predominant neutralizing antibody-inducing regions, such as S1 or RBD regions. Peptide-based vaccines that utilize evolutionarily conserved viral antigenic determinants can circumvent ADE by mitigating the potential effect associated with the substitution and glycosylation of certain amino acids, as well as conformational alterations of S proteins 92. Due to their superior structural conservation, S2 subunits are promising candidates for vaccine research and development. This approach may facilitate the generation of neutralizing antibodies, as well as memory B and T cells, targeting the SARS-CoV-2 S2 subunits in individuals without prior SARS-CoV-2 infection, thereby potentially eliciting a more robust cellular immune response against SARS-CoV-2 infection 163.

The primary mechanism underlying the ADE effect involves viral entry facilitated by the binding of antibodies to the cell surface FcγRs. To address this issue, a vaccine formulated with Mosaic nanospheres synthesized using 24-meric ferritin has been developed. This vaccine mimics the nucleotide analog structure, thereby simulating the viral entry process and avoiding ADE occurrence 164. Additionally, a nanovesicle therapy derived from CAR-T cells, which targets the SARS-CoV-2 S protein, employs a similar strategy to avoid ADE 165. Many SARS-CoV-2 vaccine candidates employ nanocarriers for the encapsulation of the vaccine components, thereby enhancing antigenic response efficacy and augmenting stability. The materials utilized in the fabrication of nanoparticle-based vaccines encompass self-assembling viral structures (virus-like particles, VLPs), lipids (including liposomes), proteins, metals, and polymers. These nanoparticles exhibit highly stability and resistance to degradation 42. Laczkó D *et al.* 75 investigated two nanoparticle-encapsulated SARS-CoV-2 mRNA-LNP vaccines: one encoding the full-length SARS-CoV-2 S protein with the furin cleavage site deleted, and the other encoding the RBD of S protein. Both vaccines elicited robust response from long-lived plasma cells (LLPCs) and memory B cells (MBCs), and they rapidly generated neutralizing antibodies that remained elevated for at least nine weeks following immunization. Under *in vitro* experimental conditions, neither of the mRNA-LNP vaccines induced ADE activity. Zhao H *et al.* 57 developed a mRNA-LNP (ARCoV) vaccine encoding RBD, and no ADE was observed in studies involving NHP. Except for mRNA vaccines, McKay PF *et al.* 166 demonstrated a vaccine that encapsulated self-amplifying RNA (saRNA) with lipid nanoparticles, which required a lower dose compared to mRNA vaccines, was capable of elicit robust antibodies and cellular responses. Importantly, no ADE phenomenon was observed *in vitro* studies. Consequently, nanoparticle-based vaccines may represent an effective strategy to mitigate the risk of ADE in SARS-CoV-2 vaccination efforts.

The focus of vaccine development may shift towards the creation of antibody-independent SARS-CoV-2 T-cell vaccines 47. Modulating the T-cell response is crucial in managing inflammation and immunopathology associated with coronavirus infections. The long-lasting virus-specific T-cell responses elicited by SARS-CoV and MERS-CoV suggest the potential for SARS-CoV-2 infection to induce long-term memory T cells 42. BNT162b1, a nucleoside-modified mRNA vaccine encapsulated in lipid nanoparticles, elicits a robust CD4^+^ T-helper 1 cell (Th1) biased immune response and a potent neutralizing antibody response by targeting RBD 167,168. Individuals vaccinated with BNT162b1 exhibited elevated levels of anti-RBD IgG antibodies compared to those who naturally infected, and demonstrated the capacity to neutralize pseudoviruses expressing various SARS-CoV-2 S protein variants 168. Similarly, the mRNA-1273 vaccine, which is encapsulated in lipid nanoparticles, encodes a full-length S protein that remains stable prior to fusion. The formulation is capable of eliciting a CD4^+^ Th1 biased immune response and generating high levels of neutralizing antibodies 168. Consequently, Hasan A *et al.* 168 have suggested that vaccines exclusively targeting the S protein may be significantly limited their ability to induce a memory CD8^+^ T cell response, potentially compromising the long-term efficacy of the vaccine. Due to the evolution of SARS-CoV-2 variants, vaccines targeting the S protein or sub-neutralizing antibodies elicited by previous coronavirus infections may enhance viral infectivity by triggering ADE, potentially leading to severe illness. Consequently, the development of mutation-specific S proteins, along with ORF1ab genes or nucleocapsid proteins, could be employed to elicit higher titers of neutralizing antibodies and memory CD8^+^ T cell immunity, thereby more accurately mimicking the immune response observed in the natural infections.

Currently, no definitive viral characteristic has been identified as having a causal relationship with ADE. Moreover, various vaccine strategies exhibit differing probabilities of inducing ADE effects. Due to the challenge of ensuring the immunogenicity of antigenic epitopes with a low risk of ADE, the antibody titers can be augmented through the use of adjuvants 3,169. According to recent reports, the inactivated SARS-CoV-2 vaccine candidate OZG-3861-01 does not induce ADE concerning T-cell and B-cell responses following the incorporation of the GM-CSF adjuvant, potentially facilitating long-term immunity 170. Aluminum, known for its typical Th2 bias in subunit and inactivated vaccines, has been utilized in SARS-CoV-2 inactivated vaccines. Notably, ADE was not observed *in vitro* in cell assays of immunized sera following administration of either with the alum-adjuvanted S1 subunit vaccine or the inactivated SARS-CoV-2 vaccine 171. Recombinant vaccines based on the S1 domain elicit a more balanced Th1/Th2 immune response compared to those based on the RBD when formulated with aluminum adjuvants 172. Beta-glucan, a naturally occurring polysaccharide, interacts with macrophages to induce a specific beneficial immune response, thereby enhancing all pathways of the immune system without causing excessive activation. Thus, it is regarded as a safer vaccine adjuvant for COVID-19 that also possesses nutritional supplementation properties, with the potential to prevent the occurrence of ADE 50. In conclusion, the judicious application of adjuvants may mitigate the risk of ADE following vaccinations.

Considering the inflammatory response mediated by IgG antibodies and the potential issues associated with ADE 173, Travis CR *et al.* proposed the generation of IgA antibodies through nasal administration for immunization. Research indicates that IgA antibodies produced by nasal-associated lymphoid tissue (NALT) persist in animal models. When IgA antibodies bind to antigens, they can block the formation of complexes between IgG, IgM, and antigens, thereby preventing complement-mediated inflammation associated with these isotypes 173. Furthermore, neither intramuscular injection nor nasal mucosal administration of the recombinant adeno-associated virus-RBD (RBD-rAAV) vaccine against SARS-CoV induced ADE phenomenon. This finding offers valuable insights for the development of nasal mucosal inoculation strategies for the SARS-CoV-2 vaccine. However, it is important to note that although IgA exhibits the highest expression levels *in vivo*, its production by mucosa-associated lymphoid tissue diminishes with advancing age. Accordingly, given that the elderly constitute the most vulnerable demographic during the epidemic, an appropriate adjuvant needs to be identified for mucosal administration of the SARS-CoV-2 vaccine.

Alternative therapeutic approaches have demonstrated efficacy in mitigating the incidence of ADE. Specifically, certain convalescent plasma therapies have been shown to decrease the potential risk of ADE by delivering substantial quantities of pathogen-specific antibodies and utilizing plasma enriched with high affinity neutralizing antibodies 43. Additionally, the technique of plasmapheresis has been suggested as a means to eliminate cytokines, interleukins, antiviral proteins, γ-globulins, and all antibodies that may trigger the ADE-FcγRII pathway (endosomal/lysosomal pathway) from the plasma 174. Cocktail therapy using multiple mAbs targeting non-overlapping epitopes has been proved to be an effective treatment for alleviating ADE 175,176. Additionally, ACE2 receptor inhibitors 127 and engineered aptamers 177 present as viable alternative strategies for circumvent the ADE phenomenon.

## 5. Summary and Perspective

ADE is implicated as primary factor contributing to severe COVID-19 infections, predominantly affecting middle-aged and elderly individuals with pre-existing health conditions 178, subsequently causing high mortality rate 179. An analysis of blood samples from COVID-19 patients revealed that the titers of neutralizing antibodies and S-protein-binding antibodies in the plasma from middle-aged and elderly patients were higher compared to those in younger patients. These elevated titers were positively correlated with the severity of COVID-19 179. MIS-C associated with COVID-19 infection has been documented across various countries during the pandemic. Research indicates that the majority of MIS-C patients exhibit elevated levels of SARS-CoV-2 antibodies in their serum, although testing negative for SARS-CoV-2 via RT-PCR 180. Furthermore, studies have demonstrated that in most COVID-19 patients, seroconversion of total antibodies, including IgM and IgG, typically occurs during the second week following symptoms onset. Notably, the seroconversion of IgG is delayed and does not coincide with a rapid reduction in viral load 181,182. The generation of virus-neutralizing antibodies, particularly anti-S protein IgG antibodies, aligns with the progression of ARDS as a result of ADE 183, a phenomenon documented in previous SARS-CoV infections 182,184. Clinical evidence has demonstrated that elevated antibody titers correlate with more severe symptoms in COVID-19 patients, potentially supporting the hypothesis that ADE may exacerbate SARS-CoV-2 infection to some degree 182. It is postulated that ADE is linked to adverse reactions following vaccination, as observed in individuals with prostate cancer (PCa). Empirical evidence indicates that the S protein of human coronavirus HCoV-229E can elicit the production of antibodies that cross-react with the SARS-CoV-2 S protein in PCa patients 185. This cross-reactivity may facilitate the ADE phenomenon in viral infections 186. Furthermore, laboratory findings suggest that SARS-CoV-2 infection could be precipitated by an immune response generated from prior exposure to endemic seasonal alphacoronaviruses NL63 and 229E 187.

Nonetheless, a perspective persists that excessive concern regarding the role of ADE in SARS-CoV-2 infection may not be warranted 178,188. In contrast to DENV, which is documented to induce ADE *in vivo*, coronaviruses predominantly target human respiratory epithelial cells rather than macrophages 178. However, it has been hypothesized that anti-S protein IgG antibodies can bind to FcγR after forming a complex with the virus, thereby initiating an ADE response mediated by FcγR. Consequently, attention should be paid to macrophages, monocytes, and myeloid cells, all of which express FcγRs 16,18,19,189. There is ongoing suspicion regarding the potential role of cross-reactive antibodies in facilitating the ADE phenomenon in coronaviruses, given the frequent occurrences of cross-neutralization among viruses in the same family, such as alphaviruses 190. At present, the validation of viral ADE phenomena through sub-neutralizing antibodies is predominantly confined to *in vitro* experiments. However, due to the absence of complex immune interactions in cell culture models, the ADE phenomenon observed *in vitro* is generally regarded as having limited representative significance 188.

While conclusive *in vivo* experimental data confirming the existence of the ADE phenomenon in SARS-CoV-2 infection is currently lacking 178,187,191, a similar phenomenon has been documented in SARS-CoV. Given their structural similarities, it remains prudent to consider potential ADE issues in the formulation of treatment regimens and the development of vaccines 191. Currently, researchers have proposed an ACE2 receptor decoy solution in the treatment of COVID-19 192,193. This involves selecting an IgG4-Fc complex 80, which minimally activates FcRs, thereby mitigating the ADE effect mediated by FcRs to the greatest extent 192. Despite the hypothesis that the ADE phenomenon following SARS-CoV-2 infection complicates vaccine development 193, it is imperative that the potential for ADE reactions post-vaccination should be thoroughly considered in both vaccine design and predictive modeling 194-196. Shielding or removing the antigenic components that may induce ADE can block or interfere with the binding of virus-antibody complex to the receptor, thereby diminishing the likelihood of ADE effects. Besides, the selection of vaccine adjuvants and inactivation techniques significantly influences the efficacy of immunization 197. In order to minimize the risk of ADE and its potential adverse outcomes, continued investigation into the ADE mechanisms remains an urgent issue 198.
